# Interaction between Laser Light and Osteoblasts: Photobiomodulation as a Trend in the Management of Socket Bone Preservation—A Review

**DOI:** 10.3390/biology9110409

**Published:** 2020-11-23

**Authors:** Andrea Amaroli, Esteban Colombo, Angelina Zekiy, Stefano Aicardi, Stefano Benedicenti, Nicola De Angelis

**Affiliations:** 1 Department of Orthopaedic Dentistry, First Moscow State Medical University (Sechenov University), 119991 Moscow, Russia; zekiy82@bk.ru; 2 Laser Therapy Centre, Department of Surgical and Diagnostic Sciences (DISC), University of Genoa, 16132 Genoa, Italy; esteban.colombo92@gmail.com (E.C.); stefano.benedicenti@unige.it (S.B.); n.deangelis74@gmail.com (N.D.A.); 3 Department for the Earth, Environment and Life Sciences (DiSTAV), University of Genoa, 16132 Genoa, Italy; stefano.aicardi94@libero.it

**Keywords:** low-level laser therapy, phototherapy, photobiomodulation, laser therapy, socket preservation, alveolar, ridge, bone augmentation, bone healing, osteoblasts

## Abstract

**Simple Summary:**

Dental implants are becoming an accepted tool, and thousands of implants are placed every year by specialists and general practitioners. However, more than 10% of bone surgeries and related procedures can show healing complications as a consequence of infections, tissue damage, or inadequate blood supply. In particular, a deficient blood supply impacts on the optimal healing process because of altered oxygen delivery to cells in the wound and a decrease in their energy supply. Researchers showed how red and infrared light affects key cellular pathways by interacting with specific photoacceptors located within the cell, particularly in mitochondria. Low-level laser therapy or photobiomodulation (PBM), as the recent medical subject heading defines it, is based on a light–cell interaction, which modifies cell metabolism by increasing oxygen consumption and ATP production through mitochondria. Although not all aspects of this interconnection are completely described, many in vitro and in vivo studies showed the benefit of PBM in wound defect management. For instance, treatment of bone with PBM results in a greater amount of new-formed osteoblasts and matrix, an increase in collagen synthesis, and microvascular reestablishment. In our review, we highlight the osteoblast–light interaction, and the in vivo therapeutic tool of PBM for socket preservation is discussed.

**Abstract:**

Bone defects are the main reason for aesthetic and functional disability, which negatively affect patient’s quality of life. Particularly, after tooth extraction, the bone of the alveolar process resorbs, limiting the optimal prosthetic implant placement. One of the major pathophysiological events in slowly- or non-healing tissues is a blood supply deficiency, followed by a significant decrease in cellular energy amount. The literature shows that photons at the red and infrared wavelengths can interact with specific photoacceptors located within the cell. Through this mechanism, photobiomodulation (PBM) can modify cellular metabolism, by increasing mitochondrial ATP production. Here, we present a review of the literature on the effect of PBM on bone healing, for the management of socket preservation. A search strategy was developed in line with the PRISMA statement. The PubMed and Scholar electronic databases were consulted to search for in vivo studies, with restrictions on the year (<50 years-old), language (English), bone socket preservation, and PBM. Following the search strategy, we identified 269 records, which became 14, after duplicates were removed and titles, abstract and inclusion-, exclusion-criteria were screened. Additional articles identified were 3. Therefore, 17 articles were included in the synthesis. We highlight the osteoblast–light interaction, and the in vivo therapeutic tool of PBM is discussed.

## 1. Introduction

Regeneration of bone tissue after oral and maxillofacial surgery reconstruction remains a challenge in medicine and dentistry. Bone defects are the main reason for aesthetic and functional disability and negatively affect a patient’s quality of life. A primary objective after bone tissue surgery is to restore the natural morphology and function of the impacted region [1,2,3]. Bone and bone-substitute grafts are, in different ways, the gold standards for bone grafting, due to their peculiar characteristics in containing bone matrix proteins and osteogenic cells, which support bone growth [3,4]. However, they might show postoperative complications, which are seen in at least 30,000 patients per year worldwide [2]. In the dentistry field, dental implants are more commonly accepted tools, and thousands of implants are placed every year by specialists and general practitioners. However, more than 10% of bone surgeries and related procedures can have healing complications as a consequence of infections, tissue damage, or inadequate blood supply and cell energy default, which lead to the alveolar bone reabsorption after tooth extraction [5].

Light at red and infrared (IR) wavelengths can affect key cellular pathways of life-forms. Indeed, in a previous review [6], we highlighted that the interaction between the non-plant cell and red/IR light wavelengths, results in an ambiguous cellular communication pathway, which is mediated by ATP, ROS, or calcium, and which leads to the manipulation of energetic cellular metabolism; this is also known by the term photobiomodulation (PBM), formerly known as low-level laser therapy (LLLT). PBM can, in this way, modulate tissue inflammation, stimulate growth factor expression and cell proliferation, and accelerate the healing processes [7]. Therefore, PBM might be a reliable method to support bone regeneration and the management of socket preservation. In this review, we explore the osteoblast–light interaction, and the in vivo therapeutic tool of PBM is discussed.

Particularly, we present a review of the literature on the effect of PBM on socket bone preservation, with the scope to point out both its in vivo effect and its support in the management of alveolar bone healing.

### 1.1. The Challenge of Socket Preservation

Different patterns of bone resorption might occur after tooth extraction. It is difficult to predict the final ridge contour and dimensions because of the remodeling of alveolar tissues [8], which considerably affects oral rehabilitation with dental implants and other prosthetic tools. The socket healing process might be conceptualized as a sequence of biological steps occurring after tooth extraction, to fill the dental alveolus with bone tissue [9]. Essentially, following a tooth extraction, a defensive fight against infection occurs via polymorphonucleocyte cell migration and coagulum formation in the impacted area. Fast angiogenesis takes place and it is accompanied and followed by an osteoclastic activity, which carries out the bone breakdown to create gaps within the bone and to promote step-by-step remodeling [10,11]. After eight weeks of healing, up to a 50% reduction of vertical bone wall height and a 20% horizontal bone resorption might be experienced by the patient. Over 12 months, 50% of the horizontal width of the ridge might disappear. Basically, the literature suggests that, within the first three months, almost two-thirds of bone reabsorption takes place [9]. Bone deformities from tooth removal might be partially avoided and repaired by a procedure called socket preservation or alveolar ridge preservation [12,13]. Socket preservation consists of conservative procedures designed to maintain the volume of bone after the extraction. It helps counteract bone resorption and reduces the need for later bone augmentation, in anticipation of a fixed partial denture-pontic or implant placement. Therefore, socket preservation supports implant success and durability by minimizing bone resorption and increasing bone formation [13,14].

Many techniques, materials, and complementary therapies were described in recent years to improve socket preservation for extraction site grafting. A number of graft materials could be used [15]:

1. Autogenous Grafts: Grafts transferred from one position to another within the same individual. This type of graft comprises cortical bone or cancellous bone and marrow, and is harvested either from intraoral or extraoral donor sites.
2. Allogeneic grafts: Grafts transferred between genetically dissimilar members of the same species. Frozen cancellous bone and marrow and freeze-dried bone are used.
3. Xenogenic grafts: Grafts taken from a donor of another species.
4. Alloplastic materials: Synthetic or inorganic implant materials that are used as substitutes for bone grafts.

The action of the grafting material used could be differentiated by [16,17]:

1. Osteoproliferative action (osteogenetic): New bone is formed by bone-forming cells (osteoblasts) contained in the grafted material; this is typical of autogenous grafts.
2. Osteoconductive action: The grafted material does not contribute to new bone formation per se but serves as a scaffold for bone formation originating from the adjacent host bone; this happens with xenogenic and alloplastic grafts.
3. Osteoinductive action: Bone formation is induced in the surrounding soft tissue immediately adjacent to the grafted material; the most widely studied type of osteoinductive cell mediator is the bone morphogenetic protein (BMP) family.

The clinician, therefore, has a wide variety of graft materials with different proprieties that could be used for the clinical procedure; it is up to the clinician to choose the best material for the right case. Basically, socket preservation is done to preserve the alveolar space derived from the extraction of the tooth. The main aim of this technique is to keep the space in the alveolar socket and prevent the collapse and resorption of the bone all around. The clinician needs to choose the right graft material by evaluating the clinical condition of the alveolar socket, such as wall integrity, surgical protocol, and healing pattern. In this way, it is possible to achieve little bone resorption of the bone, which results in a minor requirement for bone regeneration in the patient [18].

### 1.2. Laser Light–Osteoblast Interaction

The light–cell interaction is well-known in plant cells, where during the first phase of chlorophyll photosynthesis (light phase), solar energy is absorbed by chlorophyll and other pigments located in the membranes of thylakoids, inside the chloroplasts [19]. However, light–cell interactions are also described in non-plant cells, such as non-photosynthesizing prokaryotic and protozoan cells and animal cells [6]. When a photon interacts with a specific photoacceptor, its energy is absorbed to generate high-energy electrons. The excited molecule can lose its energetic status in the form of heat or fluorescence emission, or the absorbed light energy can be transferred to a photosystem molecule as an excited electron or state. In this way, the photosystem converts the photon’s energy into chemical energy, thanks to the tricky process of electron transport and a proton gradient, ending with the conversion of ADP to ATP [19]. In plants, this process occurs in the chloroplast, whereas it occurs in photoacceptors in bacteria and the conversion of ADP takes place in the inner part of the cell membrane. In other eukaryotic cells, electron transport occurs in the mitochondrial respiratory chain. This close interconnection between chloroplasts and mitochondria reflects their common origin via their bacterial ancestors and the parallel and convergent evolution of endosymbiont models [20].

Particularly, Fe-protoporphyrin (heme), Fe–S clusters, and chromophore proteins with Cu^2+^ centers of complex IV in the mitochondrial inner membrane respiratory chain show suitable features to be photoacceptors. Evidence based on the literature points out that cytochrome c oxidase (complex IV) exhibits evident absorption peaks at the red (600–700 nm) and near-infrared (NIR) (760–900 nm) wavelengths (according to its precise oxidation state) [21]; complexes I and II are not affected by light in this spectrum, while at 808 nm, complex III is poorly stimulated [22]. However, by increasing the wavelength to 1064 nm, the photon and mitochondrial complex interaction changes and while the I, III, IV, and V complexes are affected, the extrinsic mitochondrial membrane complex II and mitochondrial matrix enzymes seem to not be receptive to photons at this wavelength [23]. It is, however, of interest to take into account that other photoacceptors can exist and be involved in the ATP-, ROS-, and calcium-dependent cellular pathways, following red and IR light stimulation, such as water, transient receptor potential-V cation channels, and cellular membranes [6,24,25]. Consequently, photons can affect animal cell behavior; this medical subject heading is defined as PBM, previously known as low-level laser therapy.

However, the mechanisms through which PBM works are multifaceted and are involved in versatile biological actions such as gene expression, energy metabolism, cell proliferation, differentiation, survival, and cell death (please see review [6] and the cited literature). The fate of PBM therapy is closely related to a wide range of parameters that can be applied, such as wavelength [unit of measurement (u.m.) = nanometers (nm)], energy [u.m. = Joule (J)], fluence, i.e., the optical energy per unit area at some location (u.m. = J/cm^2^), power [u.m. = Watt (W)], power density (power per unit area. u.m. = W/cm^2^), pulse, or continuous mode of irradiation, treatment duration (s), and repetitions [26].

Concerning the in vitro approach on bone, the best-studied wavelength range, 600–700 nm, clearly showed that pre-osteoblast proliferation and viability could be positively affected [27,28,29,30,31] or not influenced [32,33,34,35,36,37] by PBM, depending on the specific laser therapy parameters employed. Particularly, the viability and the proliferation of pre-osteoblast cells were affected by an increase in matrix metalloproteinase (MMP-2) activity and activation of the extracellular signal-regulated kinase (ERK1/2) pathway [31], which are involved in the control of cell proliferation and cell death [38]. Similarly, pre-osteoblast differentiation homeostasis is also strongly modulated (or not affected) by differentiation markers and matrix mineralization such as fibroblast growth factor (bFGF) and insulin-like growth factor-1 (IGF-1) [28], both involved as autocrine and paracrine mediators of bone cell replication and differentiation. The IGF-induced PI3-kinase-Akt signaling cascade is essential for bone morphogenetic protein (BMP) 2, runt-related transcription factor (RUNX) [37], osteopontin [30,37], osteocalcin [36], collagen type I [29,31,36], and bone sialoprotein [27] expression. Similarly, the 800–900 nm wavelength range, which like 900–1000 nm better penetrates tissues than shorter wavelengths, positively [35,39,40,41,42] or negatively [35,36] affects both the proliferation and viability of pre-osteoblasts after PBM; in some cases, cells are not influenced [43,44]. Pre-osteoblast differentiation is likewise modulated by the 800–900 nm range PBM, by showing an increase in differentiation markers [37,39,41,42,45,46,47], a decrease [36] or no response [43,44].

Lastly, wavelengths higher than 900 nm are not extensively investigated, compared to shorter wavelengths. They can affect pre-osteoblast differentiation in vitro as well as proliferation and viability, although the phenomena was only described macroscopically [48,49,50]. However, Hanna et al. [1] clearly showed that 980 nm laser light improves pre-osteoblast viability and differentiation by inducing Wnt signaling and the SMAD2/3-β-catenin pathway. In a prospective analysis and following the review of Hosseinpour et al. [51], PBM administration showed promising results in vitro for bone tissue regeneration processes, via the upregulation of numerous osteogenic factors that facilitate bone formation.

## 2. Methods

**Figure A1:**
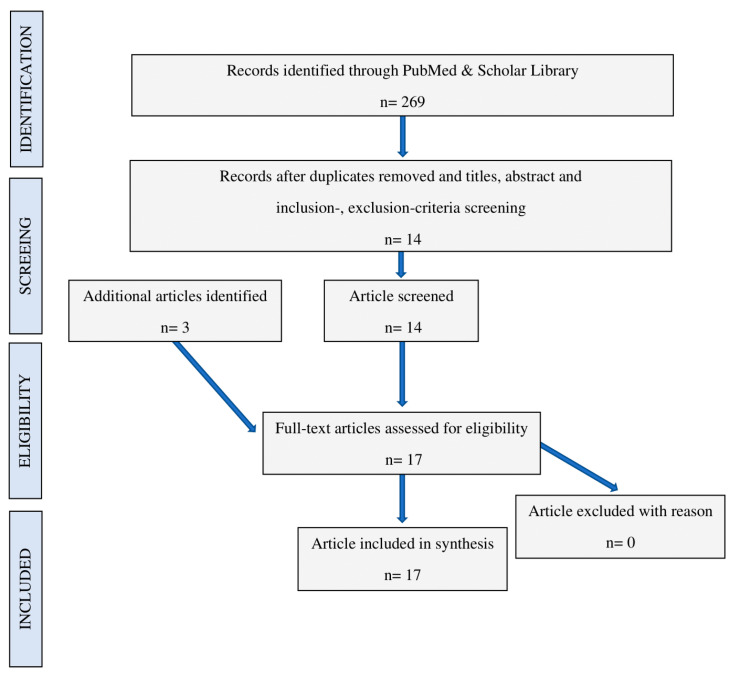
Flow chart demonstrating the selection process.

Our review was carried out in line with the PRISMA guidelines. Papers were independently searched by three authors (A.A., E.C., S.A.) on the PubMed and Scholar databases. The following keywords were applied to comply with the strategy investigation: “low-level laser therapy OR photobiomodulation OR laser biostimulation OR laser phototherapy” AND “bone augmentation OR alveolar preservation OR socket preservation OR ridge preservation”. Additional studies were also identified from the references. Articles were listed and duplicates were deleted by the three authors. They also initially screened the works by title and abstracts, according to the inclusion and exclusion criteria. Inclusion criteria were—(1) studies published in English in indexed journals on Scopus or Web of Science; (2) works published between 1 January 1970 and November 2020; (3) in vivo studies on animal models or human subjects; (3) type of LED or laser employed and treatment parameters were clearly described; and (4) therapies were immediately traceable to PBM. Exclusion criteria included (1) in vitro studies; (2) LED or laser therapies not adhering to the principles of PBM; and (3) study not focused on socket preservation. The selection process is available in Figure A1 in Appendix A and the included papers are shown in Table 1 and Table 2.

## 3. Results

Seventeen out of 269 articles selected by PubMed, Scholar, and from article references were judged to be eligible for the review. Fifty-three per cent of those were performed on animals and the remainder on humans. Among the animal studies, 67% were performed using rats, 22% using rabbits and 11% on dogs. One rat group was also exposed to gamma rays and another to bisphosphonates. A group was composed of diabetic rats. Human subjects were prevalently healthy, but in one case the patients were affected by liver diseases. Additionally, 75% of the studies were performed without biomaterial or allograft support for bone regeneration; only 25% included these materials. The in vivo studies were set up prevalently with diode lasers and using wavelengths between 808 nm and 940 nm or higher; only two studies used 780, 670, and 632.8 nm. These last wavelengths did not show an effect on bone regeneration and socket preservation, while the others induced improvements. However, Mozzati et al. [52,53] pointed out that 904–910 nm diode irradiation positively affected socket inflammation but had no effect on bone in the time windows considered. The irradiation modality was heterogeneous and prevalently performed every day for more than 5 days, up to 15 days, but some experiments were designed with only three irradiations delivered on days 0, 3, and 5, after surgery. Socket preservation was prevalently (56%) evaluated by histomorphological analysis and micro-CT, while 25% assessed osteogenic protein expression and 19% used both of these methods. A schematic representation of the selected articles for the review process is shown in Table 1 and Table 2. Lastly, only 37% of the studies correctly described the laser therapy parameters in the abstract and 31% measured the actual energy delivered by the laser device; none of the studies evaluated the temperature increase during irradiation. 

## 4. Discussion

In accordance with the growing interest in bone regeneration by the scientific community, PBM could be used to support traditional medicine. Undoubtedly, as previously shown, many steps forward were made in the understanding of the interaction between light and bone cells from the first scientific description of PBM by Professor Mester [69]. However, taking into account the in vivo and in vitro literature, PBM has discordant and contradictory effects. After a careful revision of reports, Deana et al. [70] concluded that osteoblasts are susceptible to PBM, but most of the light parameters employed by different authors unfortunately had little to no influence on proliferation and very high doses had dangerous effects on cell homeostasis, while Escudero et al. [71] pointed out that many data support the positive effects of PBM on bone regeneration, by accelerating this process. More generally, Dompe et al. [7] suggested that PBM can induce cell proliferation, enhance stem cell differentiation, and improve healing and tissue repair processes. Considering the positive effect in animal studies, authors mainly described that PBM is able to modulate inflammation in bone defects and stimulate the deposition of granulation tissue and newly formed bone tissue, as a consequence of angiogenic gene expression, as well as cyclooxygenase-2 (COX2) and vascular endothelial growth factor (VEGF) expression, during the initial phase of bone healing [71,72,73]. Additionally, PBM therapy stimulates bone regeneration when organic or inorganic materials are synergically added to the bone defect. PBM on autogenous fibrin grafts in the right parietal bone of rats induced bone regeneration, and accelerated the process of integration of the graft [74]. Irradiation of titanium scaffolds in the femurs of healthy and osteoporotic rats improved and accelerated bone repair, when compared to animals not subjected to radiation [75]. Plus, in a review, Escudero et al. [71] reported that PBM, isolated or associated with a biomaterial, remarkably improved animal disease associated with insults, trauma, osteoporosis, or osteopenia. In different settings, however, PBM did not stimulate the bioactive properties of a biosilicate, conversely inducing the deterioration of the biomechanical properties of bone [76]. Furthermore, no reduction in inflammation or amelioration in bone regeneration was described by other authors [77,78]. Therefore, adequate PBM parameters could have clinical benefits for the procedures employed daily by dentists [79].

On the topic of our review, among the 17 papers taken into account, nine were performed using animal models [54,55,56,57,58,59,60,61,62]. However, the animals investigated were prevalently rats [54,55,56,57,58,59] and in two cases, rabbits [60,61]; a dog model was surprisingly employed only in one recent work [62].

In healthy rats, 980 nm 0.01 W, 13.95 J/cm^2^ (60 s) [54] and 904/910 nm, 0.2 W, 43.8 J/cm^2^ (60 s) [56] PBM positively affected the markers of osteoblast differentiation and also improved bone mineralization. Interestingly, to support the effect of the 980 nm therapy, diabetic rats also experienced alveolar bone healing and calcification, if irradiated at the same parameters [55].

Model rats also showed improvement in alveolar bone healing, mineralization [57], and expression of osteocalcin and osteopontin [58], when irradiated to prevent the side effects of γ-ray and bisphosphonates, respectively. Lastly, alveolar bone density of rabbit ameliorated after 808 nm or 830 nm laser light irradiation, but at extremely different fluences, 1459 J/cm^2^ vs. 30 J/cm^2^, respectively.

While, rats and rabbits are a good model for wide screening, the results on bone healing cannot be directly transferred to the human clinical situation, unlike the dog model. Rats do not have a Haversian system and rabbits have a faster healing rate than humans, although a Haversian system is present. Conversely, the dog model has a human-like bone structure, dentoalveolar architecture and Haversian system [80]. Dog bone composition, density, and quality are very similar in weight, hydroxyproline content, extractable proteins, water fraction, organic fraction, and volatile inorganic fraction to human bone [80]. Bone composition and structure can directly interact with diode laser light and influence the results of therapy. In particular, laser light resonates with the vibrational overtone of water at 980 nm [81], and water appears to be a photoacceptor for this wavelength [6] or higher, influencing both the absorption of photon energy and the thermal effects. Therefore, these macro and micro-structural features and the differences in them, impede the interpretation of results from rats and rabbits, in view of translating this knowledge to the clinical setting.

For these reasons, only the work on dog models [62] is of particular interest and suggest a clinical investigation with those parameters.

During the experiment, the authors monitored the thermal increase during the irradiation. Temperature could indeed increase when higher wavelengths are irradiated and then confound the PBM’s effect. Additionally, therapy parameters were clearly described and measured by a power meter. Essentially, the bone density of extraction sockets, evaluated at 3-, 4-, and 5-weeks using cone-beam computed tomography, improved by irradiation with 980 nm diode-laser (0.60 W, 0.77 W/cm^2^, 36 J, 46 J/cm^2^, 60 s) in a continuous-wave mode [62]; both the buccal and lingual sides were irradiated to reach a total irradiation time of 120 s. In vivo animal work seem, therefore, to indicate the effectiveness of the wavelength of 980 nm, despite the parameters used in rat and dog studies being pretty far apart.

Seven [52,53,63,64,65,66,67] out of eight reviewed studies performed on human subjects described improvement on socket healing by PBM. Particularly, five studies point out the modulation of bone regeneration by laser therapy [63,64,65,66,67], two of these were in the presence of allograft and biomaterial [66,67], and only two showed an anti-inflammatory effect [52,53]. As an exception, one study revealed that PBM did not affect socket preservation [68]. Mozzati and collaborators [52,53], however, despite an adequate experimental approach, examined the effect only a few days after surgery and the therapy administered by Kucerova et al. [68] used 670 and 632.8 nm as the therapeutic wavelengths. Indeed, longer time windows (from 40 days to 4 months) allowed researchers to observe the new bone formation and bone density improvement [62,63,64,65,66,67,68]. In particular, in accordance with Schropp [9], studies with at least 8 weeks of follow-up could better describe the effect of PBM on bone healing and its possible resorption. Concerning the wavelengths, it is not strange that the only two studies [61,68] that showed no effect of PBM in animal and human models used wavelengths below 800 nm. It is known that wavelengths in the range of 800–980 nm allow better tissue penetration [82,83] than lower and higher ones, and can reach a deeper therapy target, such as bone. Indeed, wavelengths in the range of 600–700 nm showed interesting results in vitro but limited applicability in vivo. Additionally, an operational definition of PBM suggests that no significant increase in target tissue temperature is induced. Therefore, according to the absorption curves of water and the wavelengths considered in our paper, moving from the 632 nm to 1064 nm and 10,600 nm, the temperature needs monitoring and drastic increment, due to incorrect power and energies, which must be avoided [82]. In an attempt to extrapolate possible therapeutic support for socket preservation, thanks to the analysis of the devices employed, we could conclude that PBM delivered by LED or diode, Nd:YAG, and CO_2_ lasers, can positively affect alveolar ridge retention, independent of the laser used.

Unfortunately, the heterogeneity of laser parameters and the discordant, and in some cases unclear therapeutic procedures as well as the unspecified features of the probe, hand-piece, or fiber employed for laser administration, did not allow for an intergroup analysis of the studies. A major problem in PBM, as carefully reported by Tuner and Jenkins [84], is the lack of proper reporting of the parameters, which does not enable reproducibility of the experimental results. Previously, we showed [85] that fluence per se did not predict the photobimodulatory effect of laser light on the cell, but a precise relation exists between laser power and duration of exposure.it was found that ”the energy has two-component power and time of exposure, and it has been demonstrated that there is not necessarily reciprocity between them and expected effects. So, if the power doubled and the time is halved, then the same energy is delivered but a different biological response is often observed” [85]. This clearly supports the need for a description of the laser parameters as a mandatory effort to promote PBM as a clinical therapy and not only as promising experimental evidence. Additionally, the features of the probe used to deliver laser therapy could dramatically influence therapeutic success. Standard probes, hand-pieces, and fibers often have a Gaussian irradiation profile with respect to hand-pieces with a flat-top profile, influencing the energy distribution at the target and, thus, the PBM event [22]. In a previous work, we showed that if irradiation is performed with the standard hand-piece, the laser parameter setup on the laser device did not necessarily describe the precise power reaching the cell at a non-contact distance, with respect to a hand-piece with a flat-top profile [1].

The pooled data revealed the strength and limits of the use of PBM on socket preservation. The strengths were the reliable data on the osteoblast–light interaction, which highlighted the cellular pathways involved, and the energetic support of photon energy to metabolic cellular homeostasis, and thus, the healing process. The limits of PBM rest on the poor quality and the low number of studies, as well as the heterogeneity and description of the experimental set-up.

## 5. Conclusions

In conclusion, when irradiated using the appropriate parameters, PBM could improve osteoproliferation and osteoinduction for socket preservation in healthy and sick animal models and human subjects, as well as in the presence or not of an allograft or biomaterial. Wavelengths higher than 800 nm and irradiation longer than three applications, resulted in a better bone healing effect. However, the review of the current literature did not allow an unambiguous definition of therapy and suggests improving experimental set-up and performing more extensive randomized controlled trials as mandatory efforts to develop reproducible clinical therapies.

## Figures and Tables

**Table 1:** Study on photobiomodulation (PBM) and socket preservation in animal models. A schematic representation of the experimental set-up and results.

Model Employed	Teeth Extracted	Wavelength and Device	Parameters Irradiated	Therapy Administered	Methods of Detection	Effect of PBM
Rat [54]	First molars	980 nm diode with 300 μm fiber	Power = 0.01 W; Energy 0.6 J; Power density = 0.23 W/cm^2^; fluence = 13.95 for 60 s of irradiation; irradiation time: 60 s or 120 s or 300 s; continuous wave mode of irradiation (CW); spot size area = 0.043 cm^2^	For 3 and 7 days, daily	Observation period = 3 and 7 days Real-time PCR: Runx2, Col-1, osteocalcin, PDGF-B, VEGF	improvement
Rat [55] with diabetes	First molars	980 nm diode with 300 μm fiber	0.01 W; 0.23 W/cm^2^; 13.95 J/cm^2^; 60 s; CW; 0.043 cm^2^	from 3 to 14 day, daily	Observation period = 3,5,7,14 days Real-time PCR: Runx2, collagen type 1, osteocalcin, GAPDH/ Histomorphometric analyses	improvement
Rat [56]	First molars	904-910 nm diode with 5.9 mm probe	0.2 W; 0.73 W/cm^2^; 43.8 J/cm^2^; 60 s; pulsed mode of irradiation (PM); 30 kHz; 0.087 cm^2^	For 3 and 5 days, daily	Observation period = 3 and 7 days Real-time PCR: Col-1, Alp, Runx2, osteocalcin, and BMP-2; PCNA-positive cells/Micro-CT analysis/ Histomorphometric analyses	improvement
Rat [57] treated with γ-ray	First molars	830 nm diode with 18 mm probe	75 mW; CW;	Not clearly documented	Observation period = 3,7,10 days Histomorphometric analyses	improvement
Rat [58] treated with bisphosphonates	First molar (left)	1064 nm Nd:YAG with 320 μm fiber	1.25 W; 268.8 W/cm^2^; 14.37 J/cm^2^; 300 s; very short pulsed mode of irradiation, 15 Hz	For 6 day, every other day	Observation period = 8 days Western blot analysis: osteocalcin; osteopontin	improvement
Rat [59]	First molar (left)	10,600 nm CO^2^ with laser tip	1 W; 55 W/cm^2^; 40 J/cm^2^; 15 s; PM; 0.018 cm^2^	The day after surgery	Observation period = 3 and 7 days Histomorphometric analyses	improvement
Rabbit [60]	First premolars	808 nm diode irradiation probe = no specified	0.9 W; 5 W/cm^2^; 1459 J/cm^2^; 300 s; CW; 0.18 cm^2^	Immediately and every 72 h for 12 days	Observation period = 7,14,30,45 days Histomorphometric analyses	improvement
Rabbit [61]	Low inferior first premolar (right)	830 nm LED; 780 nm diode irradiation probe = no-specified	830 nm = 30 J/cm^2^; 150 s; CW 780 nm = 30 J/cm^2^; 50 s; CW	48h after surgery and then 9 irradiations	Observation period = 90 days Evaluation of impacted area (infection, hyperemia, oedema) Micro-CT analysis	830 nm = improvement 780 nm = no-effect
Dog [62]	Third premolar	980nm diode laser with flat-top hand-piece	0.60 W; 0.77 W/cm^2^; 36 J; 46 J/cm^2^; 60 s; CW	Immediately and every 48h for 14-days	Observation period = 3-, 4- and 5-weeks cone-beam computed tomography	improvement

Runx2 = runt-related transcription factor 2; Col-1 = collagen type 1; VEGF = vascular endothelial growth factor; PDGF-B = Platelet-derived growth factor B; GAPDH = glyceraldehydes-3-phosphate dehydrogenase; BMP-2 = bone morphogenetic protein 2; and PCNA = anti-proliferating cell nuclear antigen.

**Table 2:** Study on photobiomodulation (PBM) and socket preservation in human. A schematic representation of the experimental set-up and results.

Model Employed	Teeth Extracted	Wavelength and Device	Parameters Irradiated	Therapy Administered	Methods of Detection	Effect of PBM
Human [63]	First and second molars	808 nm diode irradiation probe = no specified	Power = 0.1 W; Power density = 3.6 W/cm^2^; fluence = 89 J/cm^2^; irradiation time: 25 s; continuous wave mode of irradiation (CW); spot size area = 0.028 cm^2^	Irradiated at day 0, 1, 2, 3, 4, 7, 15, in 5 points (2 vestibulars, 1 occlusal, 2 linguals)	Observation period = 45 days Micro-computed-tomography (mCT) Histomorphometric analysis	improvement
Human [64]	Lower molars	808 nm diode irradiation probe = no specified	0.1 W; 2.5 W/cm^2^; 75 J/cm^2^; 30 s; CW; 0.04 cm^2^	Irradiated at day 0, 1, 2, 3, 4, 5, 7, in 5 points (2 vestibulars, 1 occlusal, 2 linguals)	Observation period = 40 days Micro-computed-tomography (mCT) Histomorphometric analysis	improvement
Human [65]	molars	940 nm diode irradiation probe = no specified	0.9 W; 36 J; 80 s; CW	Irradiated at day 0, 1, 2, 3, 4, 7, in 2 points (1 buccal, 1 lingual)	Observation period = 56 days Histomorphometric analysis	improvement
Human [52] with hepatic disease	Molars	904-910 nm diode irradiation probe = no specified	0.2 W; 0.2 W/cm^2^; 180 J/cm^2^; 900 s; 1 cm^2^; super-pulsed (SP) 200 ns; 3 kHz	Irradiated at day 0, 3 and 5	Observation period = 7 days. Real-time PCR = IL-1B; IL-6; IL-10; Col-1; Col-3; TGF-B2; COX-2; BMP-4; BMP-7; PPAR-B	Inflammation = improved Bone = no-effect
Human [53]	molars	904-910 nm diode irradiation probe = no specified	0.2 W; 0.2 W/cm^2^; 180 J/cm^2^; 900 s; 1 cm^2^; SP 200 ns; 3 kHz	Irradiated at day 0, 3 and 5	Observation period = 7 days. Real-time PCR = IL-1B; IL-6; IL-10; Col-1; Col-3; TGF-B2; COX-2; BMP-4; BMP-7; PPAR-B	Inflammation = improved Bone = no-effect
Human [66] treated with allograft	Anterior, posterior, with roots fused teeth	Osseo-pulsed phototherapy	0.02 W/cm^2^; 1200 s	Irradiated for 21 days, daily	Observation period = 60 days Histomorphometric analysis	Improvement
Human [67] treated with biomaterial	Molars and premolars	810 nm diode	1 W; 1 W/cm^2^; 50 J/cm^2^; 50 s; 1 cm^2^; CW	Irradiated at day 0, 3, 5 and 7	Observation period = 4 months. Radiographs Histomorphometric analysis	Improvement
Human [68]	Molars	670 nm diode 632.8 nm He-Ne	670 nm = 20 mW, 1.5 J/cm^2^; 292 Hz, 9000 Hz, 20 mW, 1.5 mW; 5 Hz, 20 mW, 1.5 J/cm^2^ 632.8 nm = 5 mW, 5 Hz, 1.5 J/cm^2^	Irradiated for 4 days, daily	Observation period = 6 months. Bone density with digital radiovisiography. Protein staining = immunoglobulin A, albumin	No-effect on bone

IL = interleukin; Col = collagen type; TGF = transforming growth factor; COX = cyclooxygenase; PPAR = peroxisome proliferator-activated receptor; and BMP = bone morphogenetic protein.

## References

[1] Hanna R., Agas D., Benedicenti S., Ferrando S., Laus F., Cuteri V., Lacava G., Sabbieti M.G., Amaroli A. (2019). A Comparative Study between the Effectiveness of 980 nm Photobiomodulation Delivered by Hand-Piece with Gaussian vs. Flat-Top Profiles on Osteoblasts Maturation. Front. Endocrinol..

[2] Czerwinski M., Hopper R.A., Gruss J., Fearon J.A. (2010). Major Morbidity and Mortality Rates in Craniofacial Surgery: An Analysis of 8101 Major Procedures. Plast. Reconstr. Surg..

[3] Chaparro O., Linero I. (2016). Regenerative Medicine: A New Paradigm in Bone Regeneration. Advanced Techniques in Bone Regeneration.

[4] Sakkas A., Wilde F., Heufelder M., Winter K., Schramm A. (2017). Autogenous bone grafts in oral implantology-is it still a “gold standard”? A consutive review of 279 patients with 456 clinical procedures. Int. J. Implant Dent..

[5] Isaacson T.J. (2004). Sublingual hematoma formation during immediate placement of mandibular endosseous implants. J. Am. Dent. Assoc..

[6] Amaroli A., Ferrando S., Benedicenti S. (2019). Photobiomodulation Affects Key Cellular Pathways of all Life-Forms: Considerations on Old and New Laser Light Targets and the Calcium Issue. Photochem. Photobiol..

[7] Dompe C., Moncrieff L., Matys J., Grzech-Leśniak K., Kocherova I., Bryja A., Bruska M., Dominiak M., Mozdziak P., Skiba T.H.I. (2020). Photobiomodulation-Underlying Mechanism and Clinical Applications. J. Clin. Med..

[8] Kim D.M., De Angelis N., Camelo M., Nevins M.L., Schupbach P., Nevins M. (2013). Ridge Preservation with and Without Primary Wound Closure: A Case Series. Int. J. Periodontics Restor. Dent..

[9] Schropp L., Wenzel A., Kostopoulos L., Karring T. (2003). Bone healing and soft tissue contour changes following single-tooth extraction: A clinical and radiographic 12-month prospective study. Int. J. Periodontics Restor. Dent..

[10] Araújo M.G., Lindhe J. (2005). Dimensional ridge alterations following tooth extraction. An experimental study in the dog. J. Clin. Periodontol..

[11] Chappuis V., Engel O., Reyes M., Shahim K., Nolte L.-P., Buser D. (2013). Ridge Alterations Post-extraction in the Esthetic Zone. J. Dent. Res..

[12] De’Angelis N., Felice P., Pellegrino G., Camurati A., Gambino P., Esposito M. (2011). Guided bone regeneration with and without a bone substitute at single post-extractive implants: 1-year post-loading results from a pragmatic multicentre randomised controlled trial. Eur. J. Oral Implant..

[13] Horváth A., Mardas N., Mezzomo L.A., Needleman I.G., Donos N. (2013). Alveolar ridge preservation. A systematic review. Clin. Oral Investig..

[14] Ten Heggeler J.M.A.G., Slot D.E., Van Der Weijden F. (2010). Effect of socket preservation therapies following tooth extraction in non-molar regions in humans: A systematic review. Clin. Oral Implant. Res..

[15] Lindhe J., Lang N.P. (2015). Concept in Periodontal Tissue Regeneration. Clinical Periodontology and Implant Dentistry.

[16] Ellegaard B. (1976). Bone grafts in periodontal attachment procedures. J. Clin. Periodontol..

[17] Nielsen I.M., Ellegaard B., Karring T. (1980). KielboneR in healing interradicular lesions in monkeys. J. Periodontal Res..

[18] Fiorellini J.P., Howell T.H., Cochran D., Malmquist J., Lilly L.C., Spagnoli D., Toljanic J., Jones A., Nevins M. (2005). Randomized Study Evaluating Recombinant Human Bone Morphogenetic Protein-2 for Extraction Socket Augmentation. J. Periodontol..

[19] Simpson B.B., Niklas K.J. (1997). The Evolutionary Biology of Plants. Syst. Bot..

[20] Kream R.M., Stefano G.B., Snyder C. (2015). Mitochondria, Chloroplasts in Animal and Plant Cells: Significance of Conformational Matching. Med. Sci. Monit..

[21] Karu T.I. (1986). Molecular mechanism of the therapeutic effect of low-intensity laser irradiation. Dokl. Akad. Nauk. SSSR.

[22] Amaroli A., Ravera S., Parker S., Panfoli I., Benedicenti A., Benedicenti S. (2016). An 808-nm Diode Laser with a Flat-Top Handpiece Positively Photobiomodulates Mitochondria Activities. Photomed. Laser Surg..

[23] Ravera S., Ferrando S., Agas D., De Angelis N., Raffetto M., Sabbieti M.G., Signore A., Benedicenti S., Amaroli A. (2019). 1064 nm Nd:YAG laser light affects transmembrane mitochondria respiratory chain complexes. J. Biophotonics.

[24] Wang L., Zhang D., Schwarz W. (2014). TRPV Channels in Mast Cells as a Target for Low-Level-Laser Therapy. Cells.

[25] Ferrando S., Agas D., Mirata S., Signore A., De Angelis N., Ravera S., Utyuzh A.S., Parker S., Sabbieti M.G., Benedicenti S. (2019). The 808 nm and 980 nm infrared laser irradiation affects spore germination and stored calcium homeostasis: A comparative study using delivery hand-pieces with standard (Gaussian) or flat-top profile. J. Photochem. Photobiol. B Biol..

[26] Zein R., Selting W., Hamblin M.R. (2018). Review of light parameters and photobiomodulation efficacy: Dive into complexity. J. Biomed. Opt..

[27] Stein A., Benayahu D., Maltz L., Oron U. (2005). Low-Level Laser Irradiation Promotes Proliferation and Differentiation of Human Osteoblasts in Vitro. Photomed. Laser Surg..

[28] Saygun I., Nizam N., Ural A.U., Serdar M.A., Avcu F., Tözüm T.F. (2012). Low-Level Laser Irradiation Affects the Release of Basic Fibroblast Growth Factor (bFGF), Insulin-Like Growth Factor-I (IGF-I), and Receptor of IGF-I (IGFBP3) from Osteoblasts. Photomed. Laser Surg..

[29] Bloise N., Ceccarelli G., Minzioni P., Vercellino M., Benedetti L., De Angelis M.G.C., Imbriani M., Visai L. (2013). Investigation of low-level laser therapy potentiality on proliferation and differentiation of human osteoblast-like cells in the absence/presence of osteogenic factors. J. Biomed. Opt..

[30] Asai T., Suzuki H., Kitayama M., Matsumoto K., Kimoto A., Shigeoka M., Komori T. (2014). The long-term effects of red light-emitting diode irradiation on the proliferation and differentiation of osteoblast-like MC3T3-E1 cells. Kobe J. Med. Sci..

[31] Oliveira F.A., Matos A.A., Santesso M.R., Tokuhara C.K., Leite A.L., Bagnato V.S., Machado M.A., Peres-Buzalaf C., De Oliveira R.C. (2016). Low intensity lasers differently induce primary human osteoblast proliferation and differentiation. J. Photochem. Photobiol. B Biol..

[32] Renno A.C.M., McDonnell P., Parizotto N., Laakso E.-L. (2007). The Effects of Laser Irradiation on Osteoblast and Osteosarcoma Cell Proliferation and Differentiation in Vitro. Photomed. Laser Surg..

[33] Filho H.O.S., Reimer A.C., Marcantonio C., Marcantonio É., Marcantonio R.A.C. (2011). Effects of low-level laser therapy (685 nm) at different doses in osteogenic cell cultures. Lasers Med. Sci..

[34] Pacheco P.S., De Oliveira F.A., Oliveira R.C., Sant’Ana A.C.P., De Rezende M.L.R., Greghi S.L.A., Damante C.A. (2013). Laser Phototherapy at High Energy Densities Do Not Stimulate Pre-Osteoblast Growth and Differentiation. Photomed. Laser Surg..

[35] Ateş G.B., Ak A., Garipcan B., Yuksel S., Gulsoy M. (2015). Controversial effects of low level laser irradiation on the proliferation of human osteoblasts. Mechanisms for Low-Light Therapy X.

[36] Ateş G.B., Can A.A., Gülsoy M. (2017). Investigation of photobiomodulation potentiality by 635 and 809 nm lasers on human osteoblasts. Lasers Med. Sci..

[37] Tani A., Chellini F., Giannelli M., Nosi D., Zecchi-Orlandini S., Sassoli C. (2018). Red (635 nm), Near-Infrared (808 nm) and Violet-Blue (405 nm) Photobiomodulation Potentiality on Human Osteoblasts and Mesenchymal Stromal Cells: A Morphological and Molecular In Vitro Study. Int. J. Mol. Sci..

[38] Mosig R.A., Martignetti J.A. (2012). Loss of MMP-2 in murine osteoblasts upregulates osteopontin and bone sialoprotein expression in a circuit regulating bone homeostasis. Dis. Model. Mech..

[39] Da Silva A.P.R.B., Petri A.D., Crippa G.E., Stuani A.S., Stuani A.S., Rosa A.L., Stuani M.B. (2011). Effect of low-level laser therapy after rapid maxillary expansion on proliferation and differentiation of osteoblastic cells. Lasers Med. Sci..

[40] Li Q., Li C., Xi S., Li X., Ding L., Li M. (2019). The effects of photobiomodulation therapy on mouse pre-osteoblast cell line MC3T3-E1 proliferation and apoptosis via miR-503/Wnt3a pathway. Lasers Med. Sci..

[41] Morsoleto M., Sella V., Machado P., Bomfim F., Fernandes M.H., Morgado F., Filho G.D.J.L., Plapler H., Lopes G.D.J. (2019). Effect of low power laser in biomodulation of cultured osteoblastic cells of Wistar rats. Acta Cir. Bras..

[42] Chang B., Qiu H., Zhao H., Yang X., Wang Y., Ji T., Zhang Y., Quan Q., Li Y., Zeng J. (2019). The Effects of Photobiomodulation on MC3T3-E1 Cells via 630 nm and 810 nm Light-Emitting Diode. Med Sci. Monit..

[43] Coombe A.R., Ho C.-T.G., Darendeliler M.A., Hunter N., Philips J.R., Chapple C.C., Yum L.W.P. (2001). The effects of low level laser irradiation on osteoblastic cells. Clin. Orthod. Res..

[44] Emes Y., Akça K., Aybar B., Yalçın S., Çavuşoğlu Y., Baysal U., Işsever H., Atalay B., Vural P., Ergüven M. (2012). Low-level laser therapy vs. pulsed electromagnetic field on neonatal rat calvarial osteoblast-like cells. Lasers Med. Sci..

[45] Fujimoto K., Kiyosaki T., Mitsui N., Mayahara K., Omasa S., Suzuki N., Shimizu N. (2010). Low-intensity laser irradiation stimulates mineralization via increased BMPs in MC3T3-E1 cells. Lasers Surg. Med..

[46] Hirata S., Kitamura C., Fukushima H., Nakamichi I., Abiko Y., Terashita M., Jimi E. (2010). Low-level laser irradiation enhances BMP-induced osteoblast differentiation by stimulating the BMP/Smad signaling pathway. J. Cell. Biochem..

[47] Bomfim F.R.C.D., Sella V.R.G., Zanaga J.Q., Pereira N.S., Nouailhetas V.L.A., Plapler H. (2014). RT-PCR standardization and bone mineralization after low-level laser therapy on adult osteoblast cells. Proc. SPIE Int. Soc. Opt. Eng..

[48] Fukuhara E., Goto T., Matayoshi T., Kobayashi S., Takahashi T. (2006). Optimal low-energy laser irradiation causes temporal G2/M arrest on rat calvarial osteoblasts. Calcif. Tissue Int..

[49] Saracino S., Mozzati M., Martinasso G., Pol R., Canuto R.A., Muzio G. (2009). Superpulsed laser irradiation increases osteoblast activity via modulation of bone morphogenetic factors. Lasers Surg. Med..

[50] Medina-Huertas R., Manzano-Moreno F.J., De Luna-Bertos E., Ramos-Torrecillas J., García-Martínez O., Ruiz C. (2014). The effects of low-level diode laser irradiation on differentiation, antigenic profile, and phagocytic capacity of osteoblast-like cells (MG-63). Lasers Med. Sci..

[51] Hosseinpour S., Fekrazad R., Arany P., Ye Q. (2019). Molecular impacts of photobiomodulation on bone regeneration: A systematic review. Prog. Biophys. Mol. Biol..

[52] Mozzati M., Martinasso G., Cocero N., Pol R., Maggiora M., Muzio G., Canuto R.A. (2012). Superpulsed laser therapy on healing process after tooth extraction in patients waiting for liver transplantation. Lasers Med. Sci..

[53] Mozzati M., Martinasso G., Cocero N., Pol R., Maggiora M., Muzio G., Canuto R.A. (2011). Influence of Superpulsed Laser Therapy on Healing Processes Following Tooth Extraction. Photomed. Laser Surg..

[54] Park J.B., Ahn S.-J., Kang Y.-G., Kim E.-C., Heo J.S., Kang K.L. (2013). Effects of increased low-level diode laser irradiation time on extraction socket healing in rats. Lasers Med. Sci..

[55] Park J.J., Kang K.L. (2012). Effect of 980-nm GaAlAs diode laser irradiation on healing of extraction sockets in streptozotocin-induced diabetic rats: A pilot study. Lasers Med. Sci..

[56] Noda M., Aoki A., Mizutani K., Lin T., Komaki M., Shibata S., Izumi Y. (2016). High-frequency pulsed low-level diode laser therapy accelerates wound healing of tooth extraction socket: An in vivo study. Lasers Surg. Med..

[57] Korany N.S., Mehanni S.S., Hakam H.M., El-Maghraby E.M. (2012). Evaluation of socket healing in irradiated rats after diode laser exposure (histological and morphometric studies). Arch. Oral Biol..

[58] Mergoni G., Vescovi P., Sala R., Merigo E., Passerini P., Maestri R., Corradi D., Govoni P., Nammour S., Bianchi M.G. (2015). The effect of laser therapy on the expression of osteocalcin and osteopontin after tooth extraction in rats treated with zoledronate and dexamethasone. Support. Care Cancer.

[59] Fukuoka H., Daigo Y., Enoki N., Taniguchi K., Sato H. (2010). Influence of carbon dioxide laser irradiation on the healing process of extraction sockets. Acta Odontol. Scand..

[60] Hamad S.A., Naif J.S., Abdullah M.A. (2015). Effect of Diode Laser on Healing of Tooth Extraction Socket: An Experimental Study in Rabbits. J. Maxillofac. Oral Surg..

[61] Comunian C.R., Custódio A.L.N., De Oliveira L.J., Dutra C.E.A., Neto M.D.F., Rezende C.M.F. (2017). Photobiomodulation with LED and laser in repair of mandibular socket rabbit: Clinical evaluation, histological, and histomorphometric. Oral Maxillofac. Surg..

[62] Hamid M.A.A., Zaied A.A., Zayet M.K., Abdelmageed H., Hassan E.A., Amaroli A. (2020). Efficacy of Flat-Top Hand-Piece Using 980 nm Diode Laser Photobiomodulation on Socket Healing after Extraction: Split Mouth Experimental Model in Dogs. Photochem. Photobiol..

[63] Rosero K.A.V., Sampaio R.M.F., Deboni M., Corrêa L., Marques M.M., Ferraz E.P., Naclério-Homem M.D.G. (2020). Photobiomodulation as an adjunctive therapy for alveolar socket preservation: A preliminary study in humans. Lasers Med. Sci..

[64] Romao M.M.A., Marques M.M., Cortes A., Horliana A., Moreira M.S., LaScala C.A. (2015). Micro-computed tomography and histomorphometric analysis of human alveolar bone repair induced by laser phototherapy: A pilot study. Int. J. Oral Maxillofac. Surg..

[65] Nica D.F., Heredea E.R., Todea D.C.M. (2019). Alveolus soft and bone tissue regeneration after laser biomodulation—A histological study. Rom. J. Morphol. Embryol..

[66] Monea A., Beresescu G., Boeriu S., Tibor M., Popsor S., Antonescu D.M. (2015). Bone healing after low-level laser application in extraction sockets grafted with allograft material and covered with a resorbable collagen dressing: A pilot histological evaluation. BMC Oral Health.

[67] Lancieri L. (2011). A new bone surgical laser technique: Technical aspects and applications in dentistry. Front. Biosci..

[68] Kučcerová H., Dostálová T., Himmlová L., Bártová J., Mazánek J. (2000). Low-Level Laser Therapy after Molar Extraction. J. Clin. Laser Med. Surg..

[69] Mester E., Szende B., Gärtner P. (1968). The effect of laser beams on the growth of hair in mice. Radiobiol. Radiother..

[70] Deana A.M., De Souza A.M., Teixeira V.P., Mesquita-Ferrari R.A., Bussadori S.K., Fernandes K.P.S. (2018). The impact of photobiomodulation on osteoblast-like cell: A review. Lasers Med. Sci..

[71] Escudero J.S.B., Perez M.G.B., Rosso M.P.D.O., Buchaim D.V., Pomini K.T., Campos L.M.G., Audi M., Buchaim R.L. (2019). Photobiomodulation therapy (PBMT) in bone repair: A systematic review. Int. J. Care Inj..

[72] Babuccu C., Keklikoglu N., Baydoğan M., Kaynar A. (2014). Cumulative effect of low-level laser therapy and low-intensity pulsed ultrasound on bone repair in rats. Int. J. Oral Maxillofac. Surg..

[73] Tim C.R., Bossini P.S., Kido H.W., Malavazi I., Kress M.R.V.Z., Carazzolle M.F., Parizotto N.A., Rennó A.C. (2016). Effects of low level laser therapy on inflammatory and angiogenic gene expression during the process of bone healing: A microarray analysis. J. Photochem. Photobiol. B Biol..

[74] Gonçalves J.B.D.O., Buchaim D.V., Bueno C.R.D.S., Pomini K.T., Barraviera B., Júnior R.S.F., Andreo J.C., Rodrigues A.D.C., Cestari T.M., Buchaim R.L. (2016). Effects of low-level laser therapy on autogenous bone graft stabilized with a new heterologous fibrin sealant. J. Photochem. Photobiol. B Biol..

[75] De Vasconcellos L.M.R., Barbara M.A.M., Rovai E.S., França M.D.O., Ebrahim Z.F., De Vasconcellos L.G.O., Porto C.D., Cairo C.A.A. (2016). Titanium scaffold osteogenesis in healthy and osteoporotic rats is improved by the use of low-level laser therapy (GaAlAs). Lasers Med. Sci..

[76] Pinto K.N., Tim C.R., Crovace M.C., Matsumoto M.A., Parizotto N.A., Zanotto E.D., Peitl O., Rennó A.C. (2013). Effects of biosilicate^®^ scaffolds and low-level laser therapy on the process of bone healing. Photomed. Laser Surg..

[77] Batista J.D., Zanetta-Barbosa D., Cardoso S.-V., Dechichi P., Rocha F.S., Pagnoncelli R.M. (2014). Effect of low-level laser therapy on repair of the bone compromised by radiotherapy. Lasers Med. Sci..

[78] Acar A.H., Yolcu Ü., Altındiş S., Gül M., Alan H., Malkoç S. (2016). Bone regeneration by low-level laser therapy and low-intensity pulsed ultrasound therapy in the rabbit calvarium. Arch. Oral Biol..

[79] Kulkarni S., Meer M., George R. (2018). Efficacy of photobiomodulation on accelerating bone healing after tooth extraction: A systematic review. Lasers Med. Sci..

[80] Hillier M.L., Bell L.S. (2007). Differentiating Human Bone from Animal Bone: A Review of Histological Methods. J. Forensic Sci..

[81] Viger M.L., Sheng W., Doré K., Alhasan A.H., Carling C.-J., Lux J., Lux C.D.G., Grossman M., Malinow R., Almutairi A. (2014). Near-Infrared-Induced Heating of Confined Water in Polymeric Particles for Efficient Payload Release. ACS Nano..

[82] Fantarella D., Kotlow L. (2014). The 9.3-μm CO_2_ dental laser: Technical development and early clinical experiences. J. Laser Dent..

[83] Pandeshwar P., Roa M.D., Das R., Shastry S.P., Kaul R., Srinivasreddy M.B. (2015). Photobiomodulation in oral medicine: A review. J. Investig. Clin. Dent..

[84] Tunér J., Jenkins P.A. (2016). Parameter Reproducibility in Photobiomodulation. Photomed. Laser Surg..

[85] Amaroli A., Ravera S., Parker S., Panfoli I., Benedicenti A., Benedicenti S. (2016). 808-nm laser therapy with a flat-top handpiece photobiomodulates mitochondria activities of Paramecium primaurelia (Protozoa). Lasers Med. Sci..

